# Echocardiography in Prone Positioned Critically Ill Patients: A Wealth of Information from a Single View

**DOI:** 10.3390/diagnostics12061460

**Published:** 2022-06-14

**Authors:** Enrico Giustiniano, Sergio Palma, Massimo Meco, Umberto Ripani, Fulvio Nisi

**Affiliations:** 1 Department of Anesthesia, Intensive Care Unit and Pain Therapy, IRCCS Humanitas Clinical and Research Center, 20089 Milan, Italy; enrico.giustiniano@humanitas.it (E.G.); sergio.palma@humanitas.it (S.P.); 2 Department of Anesthesia and Intensive Care, Humanitas Gavazzeni Clinics, Via Mauro Gavazzeni, 21, 24125 Bergamo, Italy; mmeco63@gmail.com; 3 Division of Clinic Anaesthesia, Department of Emergency Hospital Riuniti, Conca Street 71, 60126 Ancona, Italy; umberto.ripani@ospedaliriuniti.marche.it

**Keywords:** point-of-care ultrasound, critical care medicine, non-invasive hemodynamics monitoring, acute respiratory failure management, imaging

## Abstract

In critically ill patients, standard transthoracic echocardiography (TTE) generally does not facilitate good image quality during mechanical ventilation. We propose a prone-TTE in prone positioned patients, which allows clinicians to obtain a complete apical four-chamber (A-4-C) view. A basic cardiac assessment can be performed in order to evaluate right ventricle function and left ventricle performance, even measuring objective parameters, i.e., tricuspid annular plane systolic excursion (TAPSE); pulmonary artery systolic pressure (PAP), from the tricuspid regurgitation peak Doppler velocity; RV end-diastolic diameter and its ratio to left ventricular end-diastolic diameter; the S’ wave peak velocity with tissue Doppler imaging; the ejection fraction (EF); the mitral annular plane systolic excursion (MAPSE); diastolic function evaluation by the mitral valve; and annular Doppler velocities. Furthermore, by tilting the probe, we can obtain the apical-five-chamber (A-5-C) view, which facilitates the analysis of blood flow at the level of the output tract of the left ventricle (LVOT) and then the estimation of stroke volume. Useful applications of this technique are hemodynamic assessment, titration of fluids, vasoactive drugs therapy, and evaluation of the impact of prone positioning on right ventricle performance and right pulmonary resistances. We believe that considerable information can be drawn from a single view and hope this may be helpful to emergency and critical care clinicians whenever invasive hemodynamic monitoring tools are not available or are simply inconvenient due to clinical reasons.

## 1. Introduction

During the COVID-19 pandemic, we observed many patients needing intensive care unit (ICU) admission and mechanical ventilation (MV) due to severe respiratory failure. In such conditions, subjects required sedation and myorelaxation to tolerate MV itself. Then, detailed cardiocirculatory evaluation and monitoring was necessary because of the effects of sedation and the high respiratory pressures. Indeed, patients under mechanical ventilation due to severe respiratory failure are often exposed to the risk of hemodynamic impairment because of continuous and even excessive intrathoracic positive pressure. Acute respiratory distress syndrome (ARDS) is one of the most frequent clinical conditions, during which the patient requires MV with a high pressure setting, aiming to re-open the collapsed and wet lung, and when it is not sufficient, the subject should be placed in prone position [1,2,3,4]. All these measures affect venous return (VR) and right ventricular performance [5].

Additionally, given the large number of patients requiring this type of care and the limited number of devices available, hemodynamic monitoring is often a logistic challenge. Consequently, cardiac ultrasound evaluation plays a crucial role in both diagnostics and monitoring, as it could be performed each time it is necessary as a noninvasive tool and then in a cost-effective way to achieve availability for all patients. Unfortunately, standard transthoracic echocardiography (TTE) generally does not allow for good image quality during mechanical ventilation. Thus, transesophageal echocardiography (TEE) has been reported to be the “gold standard” method to assess hemodynamic status [6].

## 2. Technique Proposal

In critically ill patients, the prone position may be required due to acute respiratory disease syndrome or, as in recent times, to COVID-19 respiratory failure. In our opinion, one limitation to the use of TEE in prone patients may be the position of the neck and head, which may make introduction of the TEE probe difficult and sometimes unsafe.

**Figure 1:**
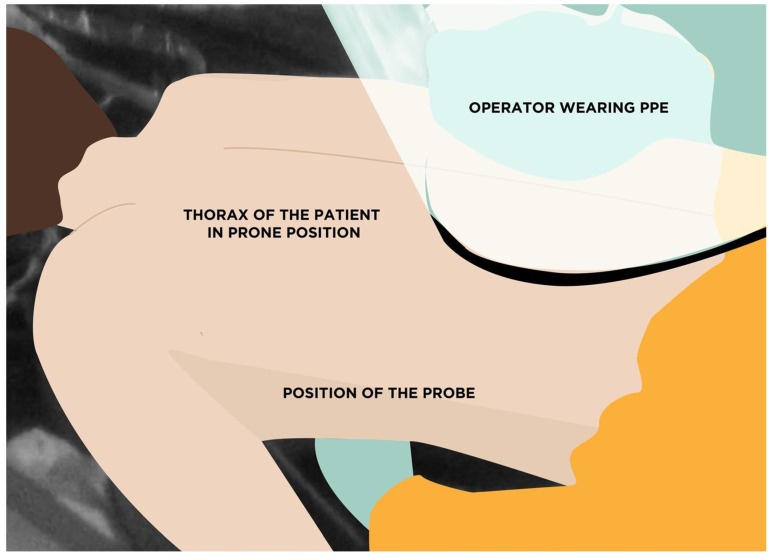
Relative positions of the operator performing echocardiography and the patient in prone position. Abbreviations: PPE, personal protective equipment. Reprinted with permission from [8].

We recently described a new proposal for TTE in prone positioned patients [7]. Our approach consisted of a temporarily deflation of the lower thoracic section of the air mattress to place the probe optimally and to obtain the apical four-chamber (A-4-C) view, taking advantage of the gravitational effect on the anatomic position of the heart within the thorax (due to the pronation), causing it to slide closer to the chest wall (Figure 1).

**Figure 2:**
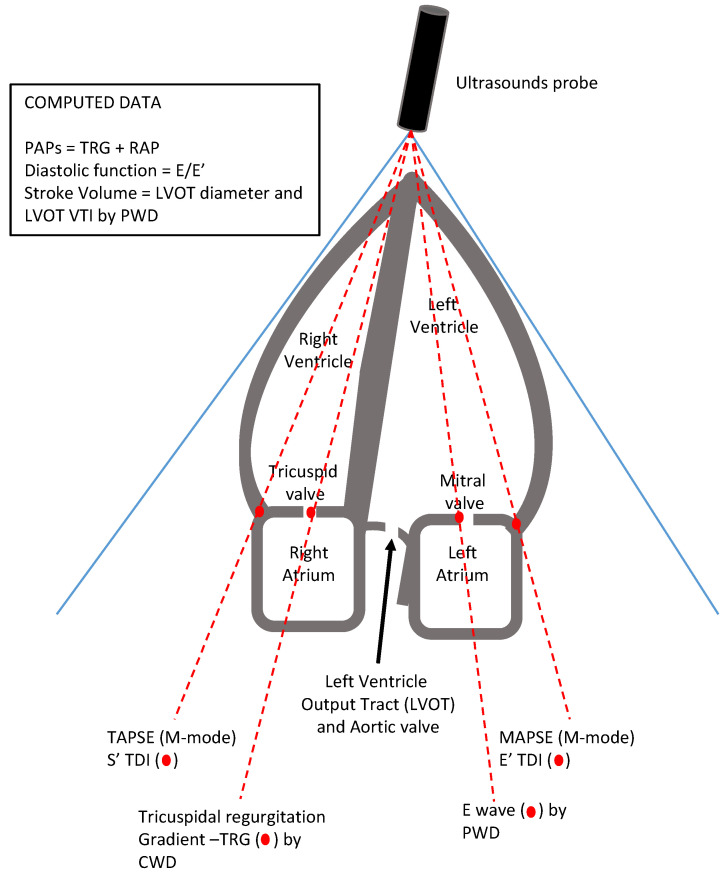
The apical-4/5-chamber views. The red points indicate where the focus of the Doppler must be placed. Abbreviations: CWD, Continuous Wave Doppler; LVOT, left ventricle output tract; MAPSE, mitral annular plane systolic excursion; TAPSE, tricuspid annular plane systolic excursion; TDI, tissue Doppler imaging; TRG, Tricuspidal Regurgitation Gradient; PWD, Pulse Wave Doppler; RAP, right atrial pressure; VTI, Velocity Time Integral.

We performed the basic TTE assessment with the same probe orientation used in supine TTE: the probe marker pointing at the patient’s right shoulder, with the probe placed at the fourth or fifth intercostal space, achieving a A-4-C view which allows right ventricle (RV) function to be evaluated, in both an “eye-ball” and parametric way. Indeed, we were able to measure tricuspid annular plane systolic excursion (TAPSE); pulmonary artery systolic pressure (PAP), computed from the tricuspid regurgitation peak Doppler velocity and the right atrial pressure (RAP) estimated by either central venous pressure or inferior vena cava distensibility index; RV end-diastolic diameter and its ratio to left ventricular end-diastolic diameter; and the S’ wave peak velocity with tissue Doppler imaging, Moreover, left ventricle (LV) performance was explored (i.e., ejection fraction (EF); the mitral annular plane systolic excursion (MAPSE); diastolic function evaluation by the mitral valve and annular Doppler velocities). Furthermore, by tilting the probe, we could obtain the apical-five-chamber (A-5-C) view, which allowed the analysis of the blood flow (the Velocity Time Integral or VTI) at the level of the output tract of the left ventricle (LVOT) and then the theoretical estimation of the stroke volume (SV) by Doppler application (Figure 2). Indeed, through the five-chamber window in this position, it is possible to obtain an adequate Doppler orientation with respect to the flow, thus avoiding any underestimation. Nevertheless, an important limitation of this approach should be taken into account. Since the only standardized measurement recommended by guidelines of the LVOT diameter is the parasternal long axis, and considering the diameter of the LVOT constant in the same patient, we suggest considering only the VTI given by the pulsed Doppler on the LVOT during hemodynamic monitoring, by using percentage changes of this (>20%) for hemodynamic monitoring of responses to fluids, vasopressors, inotropes and PEEP or recruitment maneuvers.

Even from a single view, a plethora of information about cardiac performance can be obtained with this approach. MAPSE and TAPSE (along with S’ by TDI) provide information about the systolic function of the left ventricle and right ventricle, respectively, beyond the immediate “eye-ball” evaluation of their contractility and Ejection Fraction (even the latter can also be measured). More specifically, the measurement of the left ventricle output tract, even if not validated in the A-4-C view, and the application of the pulse-wave Doppler (PWD) on the aortic valve, measuring the Velocity Time Integral (VTI), allow stroke volume to be estimated and fluid responsiveness to be evaluated, with the abovementioned limitations. The PWD on the mitral valve produces the signal of the valve flaps’ velocity during the diastolic phase. The diastolic track consists of two waves (if sinus rhythm is present): the first wave (E) represents the rapid ventricle filling; a second wave (A) represents the atrial contraction that contributes to the late filling of the left ventricle. In case of atrial fibrillation, the A-wave is lacking. Their ratio (E/A) is an a-dimensional number normally higher than 1. Moreover, the deceleration time of the E-wave can rule out a severe diastolic dysfunction when it is >150 msec in case of pseudo-normality of the E/A ratio. Conversely, an E/A ratio < 1 indicates a mild diastolic dysfunction. Finally, if we apply Tissue Doppler Imaging (TDI) on the angle between the mitral valve plane and the lateral wall of the left ventricle, with the PWD, we can obtain and measure the velocity of this portion during the diastolic phase (the velocity of the E’ wave). The ratio E/E’ is a more precise evaluation of the diastolic function of the left ventricle.

On the right heart side, if a tricuspid valve regurgitation is detected by color-mode application, continuous wave Doppler (CWD) on it allows measurement of the pressure gradient (in mmHg) between the right ventricle and the right atrium. This measurement summed to the central venous pressure gives an estimation of the systolic pulmonary artery pressure (PAPs). The central venous pressure is usually measured by a central venous catheter, which is routinely used in these patients. Alternatively, in this position, it could even be possible to obtain a transthoracic image of the IVC, placing the transducer horizontally in the right flank with the indicator pointing towards the patient’s head [9], and using measurement of the inferior vena cava diameter as an estimate of central venous pressure [10].

## 3. Literature Review

In order to validate our approach, we performed a literature search of articles published from 1 January 2019 to February 2022, through the PubMed and Scopus platforms, using keywords such as “Trans-Thoracic Echocardiography AND Prone position”, “Cardiac Ultrasounds AND Prone Position”. For the purpose of this review, articles that studied the reliability of the TTE measurement obtained in prone position have been included.

Despite the heterogeneity of the values measured by the various authors, each of them has been conducted with the purpose of demonstrating the feasibility of the TTE in prone position, both in healthy subjects and mechanically ventilated patients (Table 1). This apparent heterogeneity, however, illuminates the wide variety of possible parameters that could be measured with this innovative technique. Most of the authors cited here conducted their research on patients affected by ARDS due to COVID19. Nevertheless, we are able to provide data to support the TTE in prone position even in non-mechanically ventilated subjects.

Concerning the position used for the examination, Ugalde et al. previously described a case report about a patient receiving prone positioned TTE, but they partially modified the position of the patient using the “swimmer position” [18]. The swimmer position consists of the patient’s face facing the hand and arm that is raised at a 90-degree angle. The alternate arm should be positioned down alongside the body with the palm up. Conversely, as previously mentioned, our “trick” (i.e., the mattress sectorial deflation) allowed the physician to perform TTE without any change in the patient position, saving time and human resources for other tasks [7]. Furthermore, it avoided any potential risk associated with changing of the patient position (for example, ventilator disconnection with airdrop spreading, nasogastric tube and/or central venous lines kinking or displacement).

**Figure 3:**
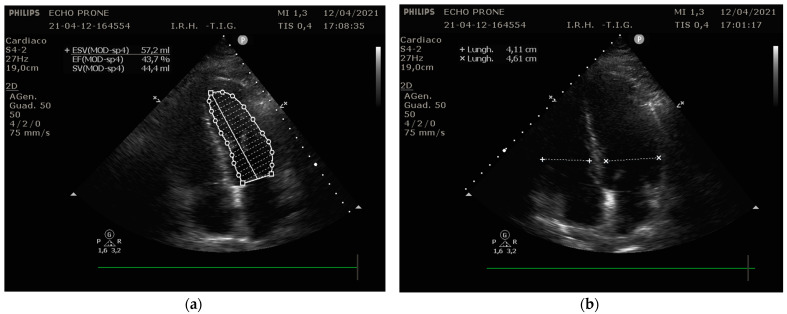
TTE apical-four-chamber views in prone position in a patient with COVID-19 ARDS. (**a**) Ejection Fraction estimation. (**b**) Ventricle diameter measurements are shown.

Focusing our analysis on the possible parameters to be recorded, Ugalde et al. [12] recently described the feasibility of the TTE in prone position, comparing the same echographic variables between two cohorts of patients—68 prone and 71 supine. They found that most of the measurements were feasible both in supine and prone position, with no difference between the two. The main difference concerned cardiac output calculation, which was less replicable in prone position. Considering that the left ventricle outflow tract was equally obtained, the limitation in prone position measurement regarded the outflow tract diameter, which required the parasternal long axis, which was not possible in that position. Conversely, left ventricle volumes and ejection fraction were more practicable in prone position because of the improved apical view in the swimmer’s position. Similar results were obtained by our group (Giustiniano et al. [8]), who described the practicability of the echo measurements through a modified prone position, as discussed before. In our case series, a single apical-four-chamber view provided sufficient data to evaluate and monitor the cardiac function in ICU (Figure 3).

Other promising evaluations were performed by Gibson et al. [12], who demonstrated that focused TTE examinations can be performed in the prone patient with ARDS, obtaining adequate echo view and adequate quantitative assessment of the RV function in 24 out of 27 patients, and adequate quantitative assessment of the LV function in 26 out of 27 patients, including morbidly obese patients and patients on high levels of PEEP, despite being supposedly technically challenging. Along the same lines, Garcia et al. [13] in their case series demonstrate that complete apical 4- and 5-chamber views and related measurements were feasible in 14 of 15 prone patients.

A novel approach was proposed by Marvaki et al. [14] and Taha et al. [17]; in fact, they used a TEE probe to obtain traditional TTE windows in prone position. Taha et al. found that all the measurements of the LVEDD, LVESD, SWT, PWT, and AoR dimensions in the prone position were accurate and replicated the corresponding measurements in the supine position well. However, it should be mentioned that a lower quality image was obtained as well as the fact that EF was lower, and LA anteroposterior diameter was smaller in the prone position if compared to supine.

Noteworthily, Santos et al. [16] focused their approach on RV function analysis in a group of spontaneous breathing healthy patients. They concluded that TTE in prone position is a useful tool to evaluate RV function; specifically, they observed that TAPSE and S’TDI were the parameters with the most superimposable results between supine and prone position.

Other interesting data were obtained by Roemer et al. [15] who studied the feasibility of the TTE in prone position among 24 young healthy patients; they found that the dedicated RV view was obtained in 100% of patients imaged, the apical four-chamber view in 95.8%, the apical long axis view in 79.2%, the apical two-chamber view in 45.8%, and the transhepatic IVC view in 33.3%; the parasternal long axis view was not visualized in any of the patients (0%). There was no statistical difference observed between the measurements recorded in the traditional and prone imaging positions.

## 4. Discussion

All the information provided by prone position TTE, along with those from biomarker measurements and semi-invasive/invasive hemodynamic monitoring, may be crucial for a complete cardiocirculatory assessment in critically ill patients, especially if under respiratory support. Indeed, in patients under mechanical ventilation, several studies reported that diastolic dysfunction is associated with a failure of mechanical ventilation weaning, both when measured by E/A and E/E’ [19]. Once again, it could be of utmost importance in a face-down recumbent subject [20]. Moreover, an ultrasound evaluation of right ventricle function during the first three days of diagnosed ARDS is recommended, also to guide the best ventilator setting, mainly in terms of positive end-expiratory pressure level [21]. Indeed, in a recent meta-analysis, RV injury in ARDS was associated with increased short-term and overall mortalities [22].

Generally, the prone position could expose the patient to the risk of cardiocirculatory failure both in the ICU (i.e., during the treatment of adult respiratory disease syndrome, ARDS) and in the operating room, mainly for spine and brain surgery, due to abrupt blood redistribution and changes in vascular pressure and resistance. Turning a patient face down is contraindicated in case of increased intracranial pressure and hemodynamic instability [18]. Hemodynamic impairment could also happen during the pronating maneuver itself or just after the patient has turned face down, mainly due to decreases in stroke volume and cardiac index because of reduced venous return [18,23].

Prone-TTE can be performed any time a hemodynamic assessment is needed and whenever there are not enough invasive hemodynamic monitoring tools available. We started to use TTE in prone position (TTEp) during the first wave of the COVID-19 outbreak in a patient requiring prone position mechanical ventilation and hemodynamic instability [7]. He was monitored only by invasive arterial line. In that case, TTEp facilitated the titration of fluids and vasoactive drugs therapy better than we could have achieved being guided only by invasive blood pressure. Then, we used TTEp in other following few cases. In a small case series, after prone positioning, we observed a reduction in PAPs and RV-End Diastolic Diameter (RV-EDD). Then, we speculated that a decrease in PAPs may be the effect of a supposed reduction in pulmonary resistance after prone positioning, as even reported by Ajam et al. [9], probably due to the reduction in the external mechanical compression of the vessels in the dorsal regions, which increases the transmural pulmonary vascular pressures in those regions. Moreover, improved ventilation of the dorsal lung regions in prone position has been thought to reduce the hypoxic vasoconstriction reflex, thus possibly contributing to lower pulmonary vascular resistances. This may translate into improved RV function as showed by the increase in S′ wave at tissue Doppler imaging (TDI) of the tricuspid valve annulus [24]. 

TDI is an echocardiographic technique that uses Doppler principles to measure the velocity of myocardial motion that is related to diastolic function. TDI is less volume-load-dependent than the traditional Doppler technique and represents the best way to evaluate the diastolic performance of the heart. Reductions in TDI tricuspid annulus systolic velocity (<15 cm/s) have been described in many severe disease settings beyond chronic heart failure, such as myocardial infarction and pulmonary hypertension [23]. As a summarizing thought, in our patients [8], right ventricle performance (affected by positive pressure mechanical ventilation) would have played a pivotal role for the outcome. If RV fails to overcome the increased afterload, mechanical ventilation may be detrimental and even life-threatening. In fact, when positive pressure respiration starts, blood venous return (and consequently the cardiac output) may fall due to lowering of the pressure gradient between the Mean Circulatory Filling Pressure (MCFP) and the Right Atrial Pressure (RAP), as the latter increases due to the rising of intrathoracic pressure [25]. Out of our small case series, the only subject who died did not show RV function improvement. Even if these findings may be speculative, they show the feasibility of performing TTE in the prone position and collecting information to guide hemodynamic management. Conversely, the effects of prone position on LV function seem to be more variable, based on the patient’s volume status. Generally, in patients with vascular depletion, the augmented intra-abdominal pressure in the prone position could lower the venous return due to the compression of the IVC, while an appreciable volume status could augment the venous return, improve RV function (as seen before) and, thus, the LV stroke volume [26].

Alongside complete echocardiographic examination, focused echocardiography protocols are being proposed to quickly assess hemodynamics in a variety of acute clinical settings. In critical care, FICE, RUSH and FATE are quite well-known [27]. Indeed, point-of-care ultrasound protocols have the advantage of being fast and, when used properly, they can give valuable information that can impact patient care. Although a complete echocardiogram is necessary for the accurate assessment of heart function, both methods are not superior to each other, but rather complementary [28]. Future trends will presumably lead to an increase in the number of protocols available and hopefully to more coherence in their approaches. To the best of our knowledge, there are no protocols to assess cardiac function in the prone position currently under study, not even a focused one. This could be the aim of future trials or study proposals.

Noteworthily, some scientific societies released their recommendations on adoption of and training in focused ultrasound aimed at emergency and critical care physicians [29,30]. The training process in focused ultrasound has been proven to be considerably fast. Indeed, Senthilnathan et al., in a prospective observational study, reported that 2 h/day of training for 30 days, allowed a group of intensivists to perform a focused trans-thoracic echocardiography with an accuracy comparable to that of cardiologists [31]. Hence, we advocate the implementation of point-of-care and/or focused ultrasound approaches in the core curriculum of emergency and critical care physicians.

Finally, beyond its diagnostic primary property, in skilled hands, TTEp may represent a valid alternative to traditional hemodynamic monitoring as it is safe, repeatable, and cost-saving, with the advantage of being available even during surges in demands of monitoring devices such as during pandemics or global health emergencies.

## 5. Limitations

Speaking of limitations of this approach, it is important to clarify that, while TAPSE and TDI are angle-independent measures, the same is not true for RV diameter and consequently right-to-left diameter ratio. As a matter of fact, RV diameter increases more and more as the probe approaches the parasternal line, resulting in an overestimation. Because of the crucial value of the RV diameter in the ventilated critically ill patients, this should be kept in mind when this measurement is obtained through TTEp.

Another limitation has already been discussed and relates to the formula used to calculate the SV, which necessitates the radius of the LVOT. This problem is overcome using VTI as a surrogate value to evaluate the fluid responsiveness in this setting in a reliable way.

## 6. Conclusions

In critically ill patients, standard transthoracic echocardiography generally does not facilitate good image quality during mechanical ventilation. We propose a prone-TTE in prone positioned patients, which allows clinicians to obtain a complete apical-four-chamber view; a basic evaluation can be performed in order to evaluate right ventricle function and left ventricle performance, even measuring objective parameters. Useful applications of this technique are hemodynamic assessment, titration of fluids and vasoactive drugs therapy, and evaluation of the impact of prone positioning on right ventricle performance and right pulmonary resistances. We believe that a wealth of information can be drawn from a single view and hope this may be helpful to emergency and critical care clinicians whenever invasive hemodynamic monitoring tools are not available or are simply inconvenient due to clinical reasons.

## Figures and Tables

**Table 1:** Comparative overview of available studies on TTE in prone position. ** Swimmer position consists of patient’s face facing the hand and arm that is raised at a 90-degree angle. The alternate arm should be positioned down alongside the body with palm up. *Modified swimmer position* uses a 10 cm-high pillow placed in the ventral infraclavicular region to allow for better transducer placement and image detection. Abbreviations: CO, cardiac output; dPWT, posterior wall thickness in diastole; dSWT, septal wall thickness in diastole; EF, ejection fraction; IVC, inferior vena cava; LA, left atrium; LV GLS, left ventricular global longitudinal strain; LVEDV, left ventricular end-diastolic volume; LVESV, left ventricular end-systolic volume; LVEDD, left ventricular end-diastolic diameter; LVESD, left ventricular end systolic diameter; LVOT-VTI, left ventricular outflow tract—velocity time integral; MAPSE, mitral annular plane systolic excursion; N/A, not available; PAPs, pulmonary artery systolic pressure; PLAX, parasternal long axis view; PSAX, parasternal short axis view; RV LS, right ventricular longitudinal strain; RV FAC, right ventricular fractional area change; RV/LV, right ventricular-left ventricular ratio; RVEDD, right ventricular end diastolic diameter; TAPSE, tricuspid annular plane systolic excursion; TDI, tissue Doppler imaging; Tr-Gr, tricuspid regurgitation gradient.

Study’s First Author	Number of Patients	BMI	Patient Position	COVID	Mechanical Ventilation	Echo Windows (and Probe)	LV Function	RV Function	Other Parameters
Ugalde et al. [11]	139 (68 in prone position)	N/A	Swimmer	Yes	Yes	-Apical 4-C -Apical 5-C (TTE phased array probe)	-LVEDV -LVESV -MAPSE -LVED area -Mitral E wave -Mitral A wave -Mitral S’ TDI -Mitral e’ TDI -LVOT-VTI -EF -CO -Septal morphology	-RVED area -TR-Gr -TAPSE -Tricuspid S’ wave	-IVC diameter
Giustiniano et al. [8]	8	N/A	Traditional Prone (with deflated air mattress)	Yes	Yes	-Apical 4-C -Apical 5-C (TTE phased array probe)	-LVEDD -LVOT-VTI -MAPSE -EF	-RVEDD -TAPSE -S’TDI -TR-Gr -RV/LV EDD	
Gibson et al. [12]	27	31 ± 5.1	Swimmer	Yes	Yes	-Apical 4-C -Apical 5-C (TTE phased array probe)	-MAPSE -Mitral E wave -Mitral A wave -e’ TDI -a’ TDI -LVOT-VTI	-TAPSE -S’ TDI -Peak TR- Gradient	
Garcia et al. [13]	15	29 ± 4.5	Swimmer	Yes	Yes	-Apical 4-C -Apical 5-C (TTE phased array probe)	-MAPSE -LVOT-VTI -E/e’ ratio -Regional wall motion abnormalities	-RVEDD -RV/LV -S’ TDI -TAPSE	
Marvaki et al. [14]	21	28 ± 4.6	Traditional prone	Yes	Yes	External TEE Probe for TTE views: -PLAX -PSAX -Apical 4-C -Subcostal SAX	N/A	N/A	(Gross estimation of LV, RV, valves function, and pericardial effusion)
Roemer et al. [15]	24	N/A	Swimmer	No	No	-Apical 4-C -Apical 2-C -Apical long axis -RV-Focused (TTE phased array probe)	-LV GLS	-RV LS -S’ TDI -RV Systolic Pressure (PAPs) -RV inflow E wave	-IVC diameter (transhepatic)
Santos-Martinez et al. [16]	50	25.65 ± 2.71	Modified Swimmer **	No	No	-Apical 4-C (TTE phased array probe)	N/A	-TAPSE -S’ TDI -RV FAC -RV End Diastolic area -RV End Systolic area -PAPs -RV/LV ratio	
Taha HS et al. [17]	30	26.4 ± 5.9	Traditional prone	No	No	External TEE probe for TTE views: -PLAX -PSAX	-LVESD -LVEDD -dSWT -dPWT -EF -LA Area -Aortic root area	(Gross estimation of RV and valves abnormalities)	
